# Growth of human tumour cell colonies from biopsies using two soft-agar techniques.

**DOI:** 10.1038/bjc.1978.165

**Published:** 1978-07

**Authors:** V. D. Courtenay, P. J. Selby, I. E. Smith, J. Mills, M. J. Peckham

## Abstract

**Images:**


*Br. J. Cancer* (1978) **38**, 77

### GROWTH OF HUMAN TUMOUR CELL COLONIES FROM BIOPSIES USING TWO SOFT-AGAR TECHNIQUES

V. D. COURTENAY*, P. J. SELBY*, I. E. SMITH†, J. MILLS* AND M. J. PECKHAM‡

*From the Divisions of *Biophysics, †Medicine and ‡Radiotherapy, Institute of Cancer Research, Sutton, Surrey*

Received 6 March 1978 Accepted 24 April 1978

**Summary.—Two techniques for growing colonies of human tumour cells in soft agar have been applied to cell suspensions derived from fresh tumour tissue from 48 patients. Colonies were obtained in 31 cases, with plating efficiencies between 0·01 and 15%. In 11 cases the plating efficiencies were 1% or above. There was evidence that some categories of tumour grew more readily than others under these conditions. The potential applications of the methods to clinical and experimental oncology are discussed.**

RECENTLY, in this laboratory, two methods have been developed for growing colonies from cell suspensions obtained from human tumours grown in immune-suppressed mice. One involves *in vitro* culture in soft agar with a replenishable liquid phase, added red blood cells and a low $O_2$ concentration (Courtenay *et al.*, 1976; Courtenay and Mills, 1978) while the other uses agar diffusion chambers (ADC) implanted i.p. into pre-irradiated mice (Smith *et al.*, 1976).

Colony assays are widely used to measure the response of established lines of animal and human tumour cells treated with cytotoxic agents, and colony formation probably provides the most reliable measure of cell survival (Roper and Drewinko, 1976) since only dividing cells believed to be capable of repopulating the tumour are measured. The techniques used in these studies have already been applied to measuring cell survival in certain xenografted human tumours treated with radiation and other cytotoxic agents. Their application to human biopsy specimens could lead to direct sensitivity testing of chemotherapeutic agents for the treatment of individual patients' tumours.

The growth of colonies directly from a solid human tumour by the *in vitro* technique has been reported by Courtenay and Mills (1978). The present paper describes the results from a series of tumours taken directly from patients and set up in agar using the *in vitro* and ADC techniques.

### METHODS

The two assay techniques have been described elsewhere in detail (Courtenay and Mills, 1978; Smith *et al*, 1976) and are presented here only in outline. The solid tumours were finely chopped using crossed scalpels and single-cell suspensions were prepared by treatment with trypsin and collagenase, or by mechanical dispersion, and filtered through a 30 $\mu$m polyester mesh to exclude clumps. Cells were examined under phase contrast using a haemocytometer, and bright cells that excluded lissamine green were counted as viable. In some tumours it was difficult to distinguish between tumour and stromal cells, and cell counts therefore included some stromal cells.

For *in vitro* growth, washed red blood cells from the rat were suspended to the original blood volume in Ham's F12 medium+15% foetal calf serum. 1 vol. of a 1/8 dilution was added to 1 vol. of tumour cells in culture medium. After adding 3 vol. of 0.5% agar medium, 1 ml aliquots of the mixture were pipetted into test tubes. When the agar had set, the cultures were incubated at 37°C in an atmosphere containing 5% $O_2$, 5% $CO_2$ and 90% nitrogen. Liquid medium was added 5 days later and changed as necessary. Colonies of more than 50 cells were counted under a dissecting microscope (×40) at 20 to 35 days. Plating efficiencies (PEs) were calculated as a percentage of the number of cells plated out.

In the ADC method, cells in similar medium and agar were introduced into Millipore diffusion chambers which were then implanted into the peritoneal cavity of C57BL mice. The mice were pretreated with 200 mg/kg of cytosine arabinoside, followed 2 days later by 900 rad of whole-body irradiation, a non-lethal combination (Millar *et al.*, 1978). The chambers were implanted within 24 h of irradiation. After removal from the mouse at about 21 days, the colonies in the ADC were scored as for the *in vitro* method.

### RESULTS

A range of different tumour types, including primary and metastatic tumours, was assayed by one or both of the techniques. In preparing cell suspensions, there were considerable variations in the numbers of suspended cells obtainable from different solid tumours. Fibrous tumours generally gave low cell yields and, in particular, 6/7 sclerous breast carcinomas treated with collagenase and trypsin yielded less than $2 \times 10^4$ single cells from about 1 g of tissue, and were therefore not set up in agar. Of the remaining solid tumours examined, 80% gave sufficient numbers of cells for testing in agar. Melanomas were found to be most readily disaggregated and, from subcutaneous deposits and lymph nodes, suspensions were obtained simply by shaking the cut-up tumour pieces in culture medium and filtering to remove the clumps. Other tumours, including all the colorectal tumours, yielded suspensions less readily, even with enzyme treatment. Ascites tumours were easier to handle, but sometimes contained large numbers of non-tumour cells. Solid and ascitic tumours were tested for growth in agar and the results are shown in the Table. Of 48 different tumours, 31 gave colonies in agar. The best results were obtained from the melanomas and ovarian tumours and colonies were grown from 12/14 and 10/10 of these tumours respectively. No colonies were obtained from 6 breast tumours. The PEs covered a wide range from 0·01 to 15%, indicating a considerable variation between individual tumours of the same type. Two of the melanomas gave PEs above 10% by one or other of the growth techniques.

Colony morphology varied with the tumour of origin, and there were differences between tumours of different categories and between individual tumours of the same category. Variation occurred in size, closeness of packing and regularity of colony outline (Fig.). Occasionally diffuse colonies were observed which were morphologically distinct from the predominant colony type, and these were not scored in the quoted PEs. Smear preparations of cells picked out from the colonies showed a morphology compatible with the tumour of origin, and melanotic melanomas gave black colonies of cells containing melanin granules.

Some of the tumours examined were also implanted directly into immune-suppressed mice and grown as xenografts. The appearance of colonies obtained for 5 xenografted melanomas, an ovarian tumour, a uterine tumour and a colonic carcinoma, when compared with corresponding colonies grown directly from the original biopsies, showed a close resemblance. Their PEs were also comparable, and tumours which gave high PE from the original biopsy usually gave good PE when grown from the xenografts.

### DISCUSSION

There have been numerous previous attempts at culturing cells directly from human tumours, with the aim of developing methods of testing the chemosensitivity of individual tumours (Hall, 1977; Dendy *et al.*, 1970) but these have been hampered by the difficulty of culturing cells taken directly from tumour specimens, and of determining whether the cells which do

TABLE.—*Plating efficiencies (PE) of colonies grown* in vitro *or in ADC from cell suspensions prepared from tumour biopsies*

| | | | PE | | | |
|---|---|---|---|---|---|---|
| | | Treatment of | Tumour biopsy | | Xenograft | |
| Tumour type | Form | suspension | *in vitro* | ADC | *in vitro** | ADC |
| Melanoma | S.c. deposit | m | 0 | 0·23 | 0·2 | 1·0 |
| | S.c. deposit | m | 15 | >2·5 | 20·0 | 15·0 |
| | S.c. deposit | m | 0·5 | 0 | 0·01 | 1·0 |
| | S.c. deposit | m | 3·0 | 0·92 | 10·0 | 11·0 |
| | S.c. deposit | m | 5·6 | 11·5 | — | — |
| | S.c. deposit | m | 0 | 0·066 | — | — |
| | S.c. deposit | m | 0·2 | — | — | — |
| | Node deposit | m | 0 | 2 | — | — |
| | Node deposit | m | 0 | — | 0·07 | 0·04 |
| | Node deposit | m | 0·5 | — | — | — |
| | Node deposit | m | 0·25 | — | — | — |
| | Primary | m | 0 | 0·6 | — | — |
| | Ascites | u | 0 | 0 | — | — |
| | Pleural effusion | u | — | 0·072 | — | — |
| Ovarian Ca | Ascites | u | 2·7 | 0·13 | — | — |
| | Ascites | u | — | 0·01 | — | — |
| | Ascites | u | 0·25 | — | — | — |
| | Ascites | u | 0·02 | — | — | — |
| | Ascites | u | 0·2 | — | — | — |
| | Ascites | u | 1·0 | — | — | — |
| | Ascites | u | 1·0 | 1·7 | — | — |
| | Primary | t | 0·4 | — | — | — |
| | Secondary dep. | t | 1·3 | — | — | — |
| | Peritoneal dep. | m | 4·5 | 2·2 | 0·55 | 3·0 |
| Breast Cancer | Primary | t | 0 | 0 | — | — |
| | Primary | t | 0 | — | — | — |
| | Primary | t | 0 | — | — | — |
| | Pleural effusion | u | 0 | 0 | — | — |
| | Pleural effusion | u | 0 | 0 | — | — |
| | Pleural effusion | u | 0 | — | — | — |
| Colorectal Cancer | Primary | t+c | 0 | — | — | — |
| | Primary | t+c | 0 | 0 | — | — |
| | Primary | t | 0·03 | 0 | — | — |
| | Primary | t | 0 | — | — | — |
| | Primary | t | — | 0·25 | — | — |
| | Secondary dep. | t+c | 0·3 | 2·0 | 0·03 | — |
| Teratoma testis | Primary | t+c | — | 0 | — | — |
| | Primary | t | 0 | — | — | — |
| Seminoma | Primaiy | t | 0 | 0·10 | — | — |
| | Primary | m | — | 0 | — | — |
| Pancreatic Ca | Primary | t+c | 0·17 | 1·0 | — | — |
| Uterine Ca (body) | Secondary dep. | m | 12 | — | 10·1 | — |
| (cervix) | Ascites | | — | 0 | — | — |
| Oat-cell Ca bronchus | Secondary dep. | m | — | 0·1 | — | — |
| Hypernephroma | Secondary dep. | t | 0·1 | 0 | — | — |
| Orchioblastoma | Primary | t | 0·04 | — | — | — |
| Osteosarcoma | Local recurrence | m | — | 0 | — | — |
| Leiomyosarcoma | Primary | m | 0 | — | — | — |

u = untreated
m = mechanical separation
t = trypsin
t + c = trypsin + collagenase
— = not done

* Xenografted tumours derived from the original biopsy were tested after the 1st or 2nd passage in immune-suppressed mice.

FIG.—Colonies grown *in vitro* and viewed in agar: a. Amelanotic melanoma ×150; b. Melanotic melanoma ×75; c. Ovarian ascites ×150.

grow are of tumour or stromal origin. Stromal cells that attach to culture dishes and grow as monolayers are unable to form colonies in agar, and the ability to produce colonies in agar (Macpherson, 1973) is one of the criteria of malignant transformation. Soft agar has extensively been used for the growth of marrow cells and some established cell lines, but there is little information regarding the growth of human tumour cells in agar without previous growth in monolayer culture, although colony formation from childhood solid tumours (McAllister and Reed, 1968; Altman *et al.*, 1975) has been reported. Recently, Hamburger and Salmon (1977*a*, *b*) have grown agar colonies from human myeloma cells and also from other malignant cells of marrow. For 3 tumours, a bronchial carcinoma, a neuroblastoma and an ovarian ascites, they obtained PEs between 0·02 and 0·1%. In the present studies we have examined 48 different solid or ascites tumours. Compact colonies of over 50 cells were obtained from over half of the tumours tested and PEs averaging 2% were measured.

Evidence that colonies were derived from tumour cells was provided by cell morphology, and was particularly clear in the case of the melanotic melanomas, but was also shown from the distinctive colony morphology and histological appearance of colonies grown from other tumour types. Although agar inhibits the formation of colonies by stromal cells that grow as monolayers, the possibility that blood cells from the tumour might be capable of producing diffuse colonies similar to those seen in marrow cultures was considered, and occasional diffuse colonies were excluded from the colony counts.

The two agar techniques used in these studies differ from standard agar techniques in providing for the replenishment of the agar medium from a liquid phase, by the addition of fresh culture medium above the agar or by growing the cells in ADC in the mouse so that nutrients are renewed by diffusion from the peritoneal fluid. It might be thought that under these *quasi*

*in vivo* conditions, cell growth in ADC would be better than *in vitro*. Although in a minority of cases better growth was obtained with one or other of the systems, there was no evidence that either of them was generally superior.

The PEs obtained from all the tumours which gave colonies covered a wide range of values (0·01 to 15%). There are probably many reasons for this. Some of the lower PEs could be related to technical problems involved in preparing cell suspensions, possibly causing irreparable damage to the cells. Also, particularly in primary tumours with a partly differentiated cell population, a considerable proportion of the tumour cells may lack the capacity for further proliferation. In other tumours, there may be essential differences in nutritional, hormonal and other requirements specific to the tumour of origin. Alternatively, in more slowly growing tumours the rate of proliferation may be too low for the production of colonies within the period of observation, since to produce a colony of over 50 cells in a period of 3 weeks requires that the cells divide at least once every $3\frac{1}{2}$ days.

In spite of the limitations outlined, colonies have been obtained from over half of the tumours tested, and for a number of them the PEs large enough to form the basis of a survival assay. Although further experience and technical development, particularly in the preparation and separation of tumour-cell suspensions, is needed, these results, particularly from the melanomas, suggest that it could eventually be possible to undertake sensitivity testing of tumours from individual patients, at least for certain defined tumour categories. Other possible clinical applications are in the estimation of response to chemotherapy, where multiple samples are available and the growth of colonies in agar could also be of prognostic value, since it gives a measure of the proliferation rate of the tumour cells. These techniques already provide a useful research tool for the clonal isolation of tumour cell lines and could potentially allow the analysis of clonal heterogeneity in tumours.

Our thanks are due to Dr G. G. Steel for his support and encouragement, to all members of the medical staff who supplied the material, including Drs J. Baker, T. J. McElwain, P. Trott and E. Wiltshaw and to the tissue collectors, Mrs J. Nicholls, Mrs J. Whittaker and Mrs S. Schonfield.

### REFERENCES

ALTMAN, A. J., CRUSSI, F. G., RIERDEN, W. J. & BAEHNER, R. L. (1975) Growth of rhabdomyosarcoma colonies from pleural fluid. *Cancer Res.*, **35,** 1809.

COURTENAY, V. D., SMITH, I. E., PECKHAM, M. J. & STEEL, G. G. (1976) *In vitro* and *in vivo* radiosensitivity of human tumour cells obtained from a pancreatic carcinoma xenograft. *Nature*, **263,** 771.

COURTENAY, V. D. & MILLS, J. (1978) An *in vitro* colony assay for human tumours grown in immune-suppressed mice and treated *in vivo* with cytotoxic agents. *Br. J. Cancer*, **37,** 261.

DENDY, P. P., BOZMAN, G. & WHEELER, T. K. (1970) *In vitro* screening test for human malignant tumours before chemotherapy. *Lancet*, ii, 68.

HALL, T. C. (1977) Prediction of responses to therapy and mechanism of resistance. *Semin. Oncol.*, **4,** 193.

HAMBURGER, A. W. & SALMON, S. E. (1977*a*) Primary bioassay of human tumour stem cells. *Science*, **197,** 460.

HAMBURGER, A. W. & SALMON, S. E. (1977*b*) Primary bioassay of human myeloma stem cells. *J. Clin. Invest.*, **60,** 846.

MCALLISTER, R. M. & REED, G. (1968) Colonial growth in agar of cells derived from neoplastic and non-neoplastic tissues of children. *Ped. Res.*, **2,** 356.

MACPHERSON, I. A. (1973) Soft agar techniques. In *Tissue Culture Methods and Applications*. Eds. Kruse, P. F. and Patterson, M. K. New York: Acad. Press. p. 267.

MILLAR, J. L., BLACKETT, N. M. & HUDSPITH, B. H. (1978) Enhanced post-irradiation recovery of the haemopoietic system in animals pre-treated with a variety of cytotoxic agents. *Cell Tissue Kinet.* **11,** in press.

ROPER, P. R. & DREWINKO, B. (1976) Comparison of *in vitro* methods to determine drug-induced cell lethality. *Cancer Res.*, **36,** 2182.

SMITH, I. E., COURTENAY, V. D. & GORDON, M. Y. (1976) A colony-forming assay for human tumour xenografts using agar in diffusion chambers. *Br. J. Cancer*, **34,** 476.


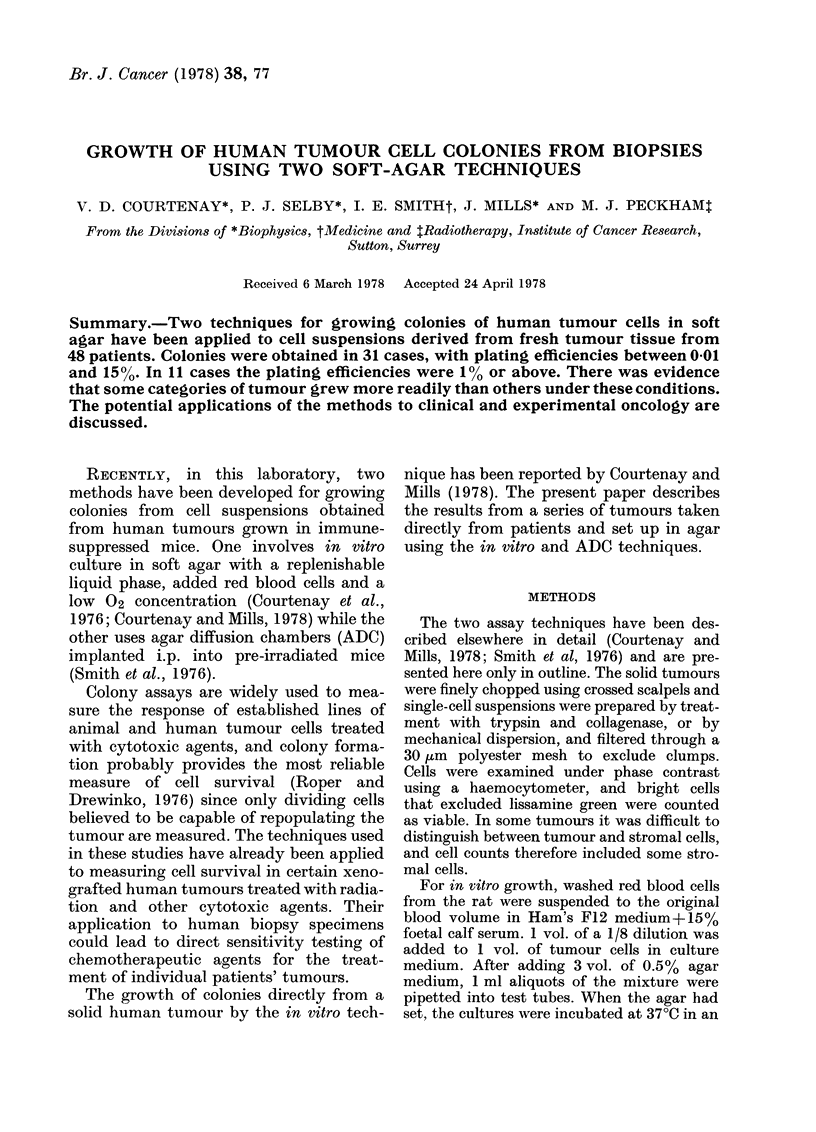

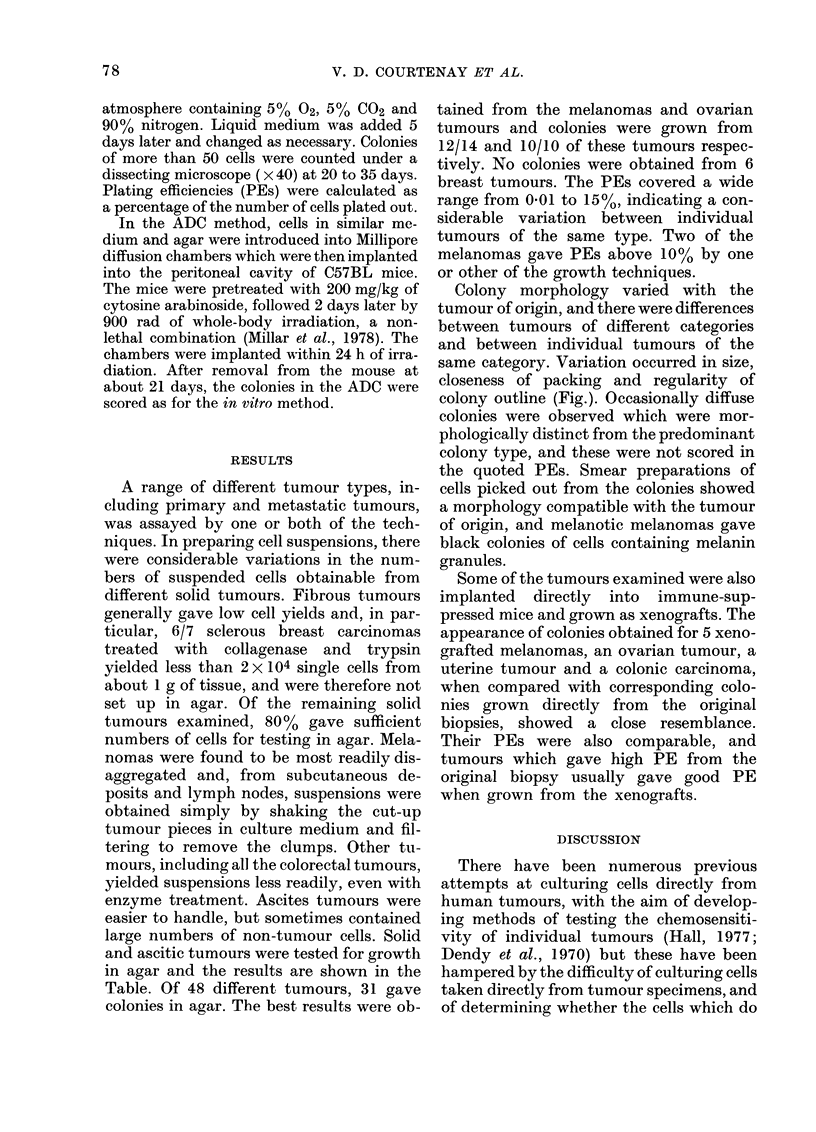

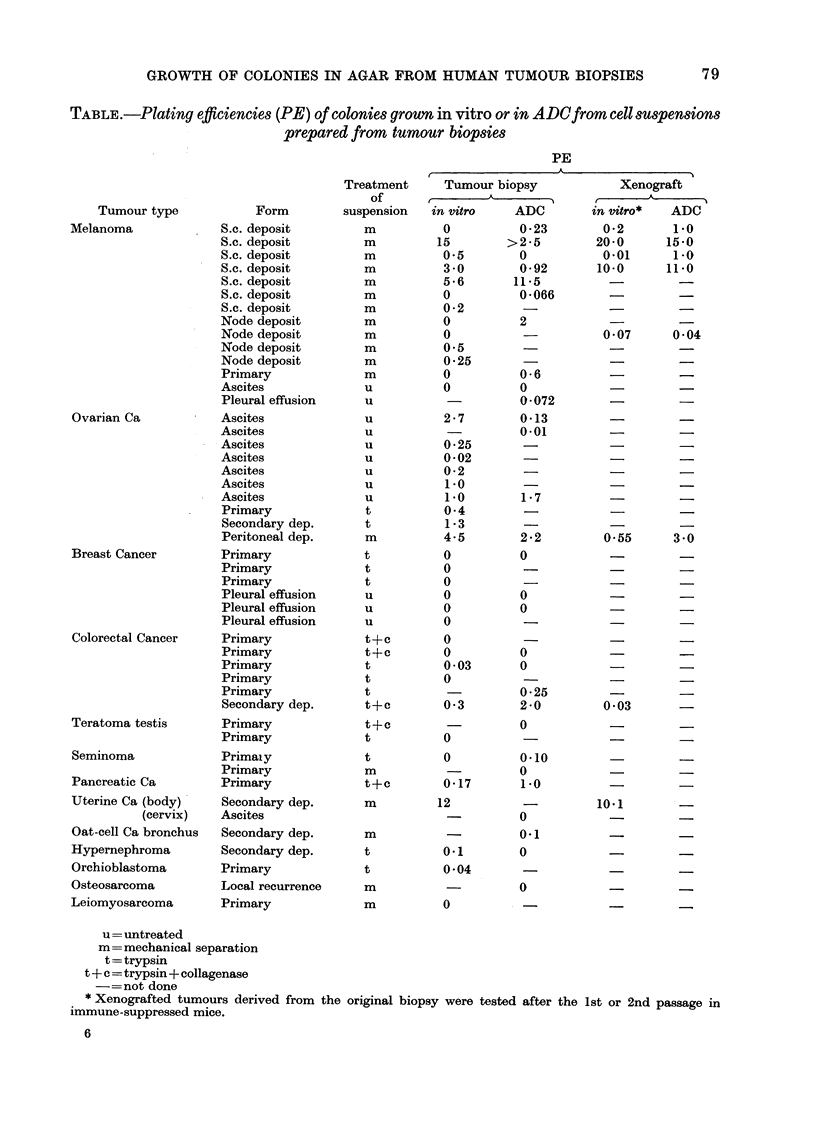

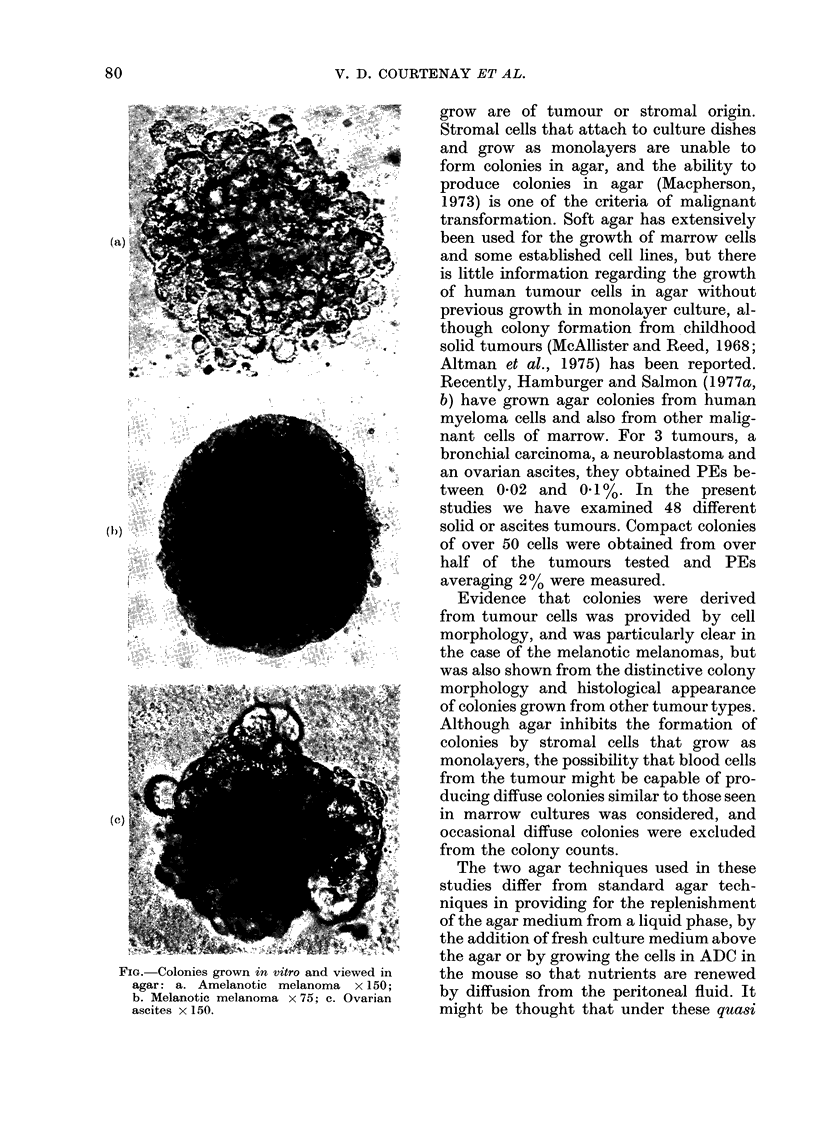

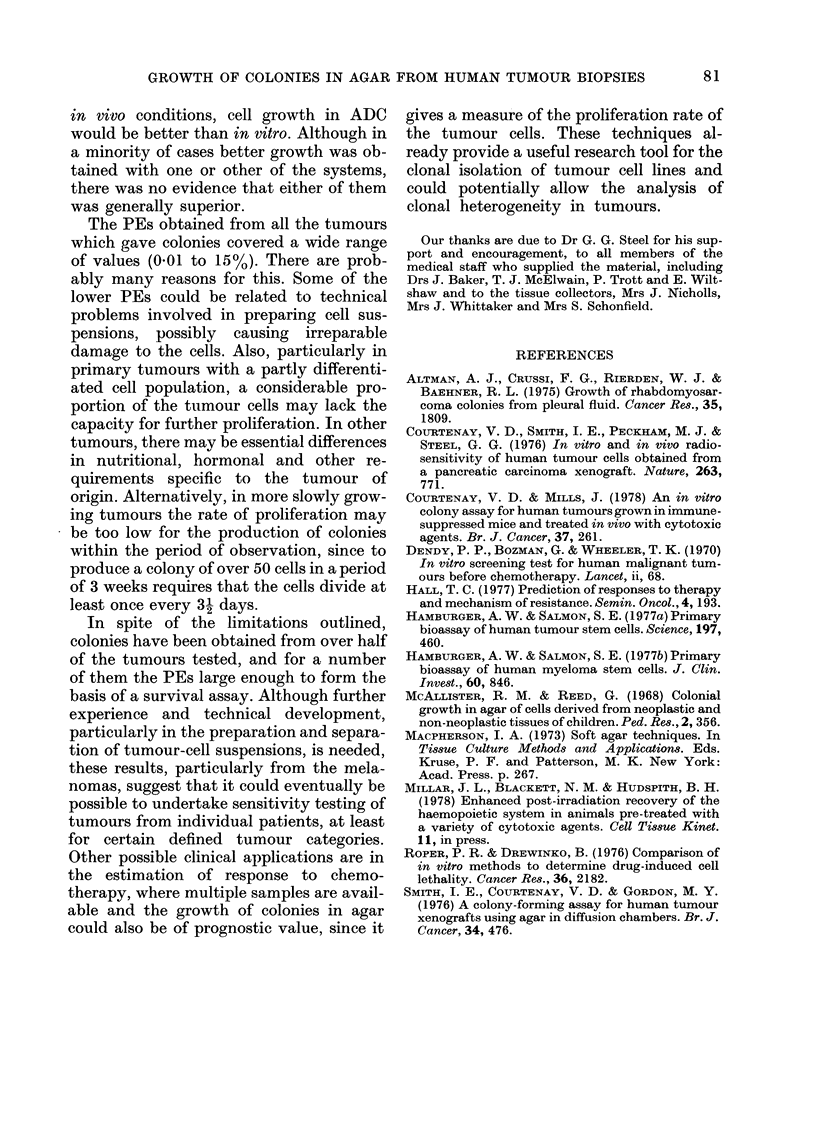


## References

[OCR_00675] Courtenay V. D., Mills J. (1978). An in vitro colony assay for human tumours grown in immune-suppressed mice and treated in vivo with cytotoxic agents.. Br J Cancer.

[OCR_00668] Courtenay V. D., Smith I. E., Peckham M. J., Steel G. G. (1976). In vitro and in vivo radiosensitivity of human tumour cells obtained from a pancreatic carcinoma xenograft.. Nature.

[OCR_00681] Dendy P. P., Bozman G., Wheeler T. K. (1970). In-vitro screening test for human malignant tumours before chemotherapy.. Lancet.

[OCR_00686] Hall T. C. (1977). Prediction of responses to therapy and mechanisms of resistance.. Semin Oncol.

[OCR_00694] Hamburger A., Salmon S. E. (1977). Primary bioassay of human myeloma stem cells.. J Clin Invest.

[OCR_00699] McAllister R. M., Reed G. (1968). Colonial growth in agar of cells derived from neoplastic and non-neoplastic tissues of children.. Pediatr Res.

[OCR_00716] Roper P. R., Drewinko B. (1976). Comparison of in vitro methods to determine drug-induced cell lethality.. Cancer Res.

[OCR_00721] Smith I. E., Courtenay V. D., Gordon M. Y. (1976). A colony-forming assay for human tumour xenografts using agar in diffusion chambers.. Br J Cancer.

